# Inhaled nitric oxide improves transpulmonary blood flow and clinical outcomes after prolonged cardiac arrest: a large animal study

**DOI:** 10.1186/s13054-015-1050-2

**Published:** 2015-09-15

**Authors:** Matthias Derwall, Andreas Ebeling, Kay Wilhelm Nolte, Joachim Weis, Rolf Rossaint, Fumito Ichinose, Christoph Nix, Michael Fries, Anne Brücken

**Affiliations:** Department of Anesthesiology, University Hospital RWTH Aachen, Pauwelsstr. 30, 52074 Aachen, Germany; Institute for Neuropathology, University Hospital RWTH Aachen, Pauwelsstr. 30, 52074 Aachen, Germany; Anesthesia Center for Critical Care Research, Department of Anesthesia, Critical Care, and Pain Medicine, Massachusetts General Hospital and Harvard Medical School, 55 Fruit Street, Boston, MA 02114 USA; Abiomed Europe GmbH, Neuenhofer Weg 3, D-52074 Aachen, Germany; Department of Anesthesiology, St. Vincenz Hospital Limburg, Auf dem Schafsberg, 65549 Limburg, Germany

## Abstract

**Introduction:**

The probability to achieve a return of spontaneous circulation (ROSC) after cardiac arrest can be improved by optimizing circulation during cardiopulomonary resuscitation using a percutaneous left ventricular assist device (iCPR). Inhaled nitric oxide may facilitate transpulmonary blood flow during iCPR and may therefore improve organ perfusion and outcome.

**Methods:**

Ventricular fibrillation was electrically induced in 20 anesthetized male pigs. Animals were left untreated for 10 minutes before iCPR was attempted. Subjects received either 20 ppm of inhaled nitric oxide (iNO, n = 10) or 0 ppm iNO (Control, n = 10), simultaneously started with iCPR until 5 hours following ROSC. Animals were weaned from the respirator and followed up for five days using overall performance categories (OPC) and a spatial memory task. On day six, all animals were anesthetized again, and brains were harvested for neurohistopathologic evaluation.

**Results:**

All animals in both groups achieved ROSC. Administration of iNO markedly increased iCPR flow during CPR (iNO: 1.81 ± 0.30 vs Control: 1.64 ± 0.51 L/min, p < 0.001), leading to significantly higher coronary perfusion pressure (CPP) during the 6 minutes of CPR (25 ± 13 vs 16 ± 6 mmHg, p = 0.002). iNO-treated animals showed significantly lower S-100 serum levels thirty minutes post ROSC (0.26 ± 0.09 vs 0.38 ± 0.15 ng/mL, p = 0.048), as well as lower blood glucose levels 120–360 minutes following ROSC. Lower S-100 serum levels were reflected by superior clinical outcome of iNO-treated animals as estimated with OPC (3 ± 2 vs. 5 ± 1, p = 0.036 on days 3 to 5). Three out of ten iNO-treated, but none of the Control animals were able to successfully participate in the spatial memory task. Neurohistopathological examination of vulnerable cerebral structures revealed a trend towards less cerebral lesions in neocortex, archicortex, and striatum in iNO-treated animals compared to Controls.

**Conclusions:**

In pigs resuscitated with mechanically-assisted CPR from prolonged cardiac arrest, the administration of 20 ppm iNO during and following iCPR improved transpulmonary blood flow, leading to improved clinical neurological outcomes.

## Introduction

Optimizing vital organ blood flow during cardiopulmonary resuscitation (CPR) has been recognized as the key element to increase the rate of return of spontaneous circulation (ROSC, primary survival) and to achieve a favorable long-term neurocognitive outcome (secondary survival) [1].

In a model of prolonged cardiac arrest we have recently shown that a percutaneous left ventricular assist device (pLVAD, Impella 2.5, Abiomed Inc., Danvers, MA, USA) can double survival [2] when used for cardiopulmonary resuscitation in place of conventional chest compressions. We coined this pLVAD-based approach intraventricular CPR (iCPR), as opposed to conventional chest compression CPR. With iCPR, the left ventricle can be unloaded using a device with similar dimensions to a catheter. The non-pulsatile bloodstream unloads directly into the ascending aorta without any additional external circulation. We proposed this technique as a less invasive alternative to extracorporeal CPR (eCPR) using extracorporeal membrane oxygenation (ECMO), as iCPR requires the cannulation of only one femoral vessel with a 13-F sheath introducer rather than two large-bore cannulas when using ECMO.

However, we observed that the achievable flow capacity during ventricular fibrillation (VF) remained lower than expected with roughly 1.36 ± 0.02 L/minute, although the iCPR-device is technically capable of delivering a maximum of 2.5 L/minute. We hypothesized that this flow restriction was primarily due to impaired left ventricular filling, caused by depressed right-to-left transpulmonary blood flow during VF [2].

Inhaled nitric oxide (iNO) was originally developed as a selective pulmonary vasodilator and has been approved since 1999 for the treatment of neonatal hypoxemia with pulmonary hypertension [3]. It is estimated that more than 400,000 Americans have been treated with inhaled NO, with an excellent safety track record [4]. Additionally, iNO has been shown to have systemic effects without causing systemic vasodilation. For example, breathing NO attenuates myocardial ischemia/reperfusion injury in mice [5] and swine [6] and hepatic ischemia/reperfusion injury in patients undergoing liver transplantation [7]. It is also of note that iNO has been shown to improve the maximum flow capacity of a surgically implanted extracorporeal LVAD in a porcine model of hypoxemia-induced pulmonary hypertension and right ventricular failure [8].

Based on these prior observations, we hypothesized that iNO would increase the maximum achievable iCPR flow capacity during resuscitation, and ameliorate the reperfusion injury following ROSC, hence leading to improved clinical outcomes after cardiac arrest. To address this hypothesis, we examined the effects of administering iNO at 20 ppm during and following iCPR in a swine model of prolonged cardiac arrest. Here, we report that iNO markedly increased the flow capacity of iCPR and improved the neurological outcome after cardiac arrest.

## Methods

A large-animal model of cardiac arrest and CPR was employed, as has been previously described by our group [2, 9–12]. Twenty male domestic pigs at 4 months of age (44.1 ± 5.8 kg) were studied. All procedures were conducted in accordance with the principles for the care and use of animals based on the Helsinki Declaration, and approved by the appropriate governmental institution (Landesamt für Natur, Umwelt und Verbraucherschutz NRW, LANUV, Recklinghausen, Germany).

### General preparation

Following induction of anesthesia, animals were intubated and equipped with percutaneous catheters and sheath introducers in femoral arteries and veins before a 5-French pacing catheter was placed in the cephalic vein. Animals were then equipped with a modified Impella 2.5 (iCPR-device) with a shortened angled cannula as described in [2]. All animals received two intravenous (IV) injections of 5,000 IU heparin and 1.5 g of cefuroxime to prevent blood clotting and wound infections.

### Experimental protocol

**Fig. 1:**
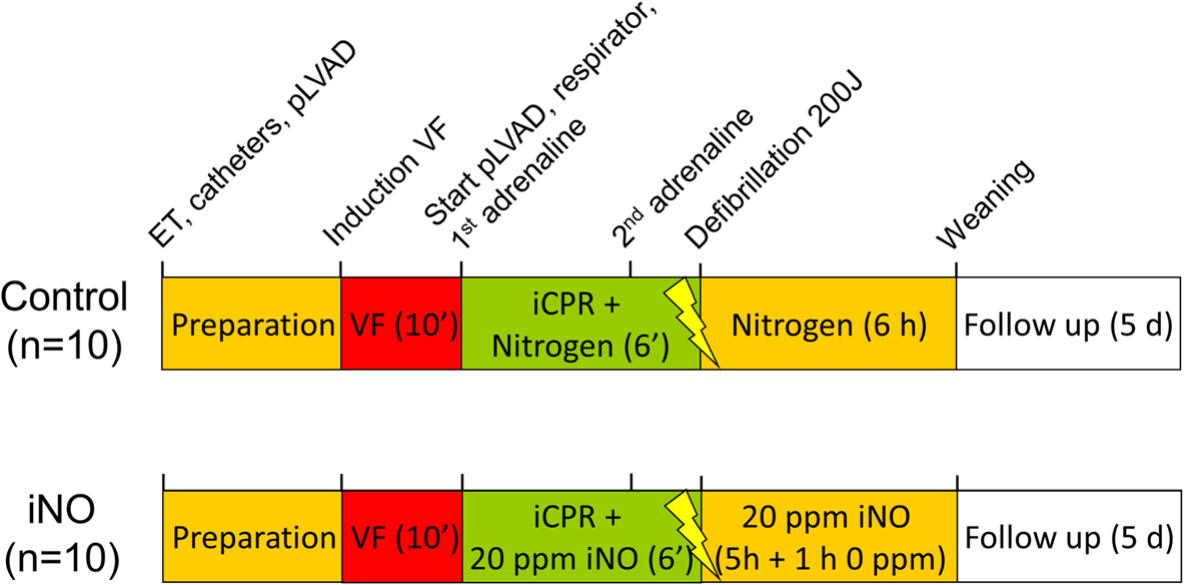
Experimental procedure. Flowchart depicting the sequence of actions taken in the experiments comparing animals treated with 20 ppm inhaled nitric oxide (iNO, n = 10) to control (n = 10). Note that no external chest compressions were applied during any stage of the experiment. *ET* endotracheal tube, *pLVAD* percutaneous left ventricular assist device, *VF* ventricular fibrillation

Please see Fig. 1 for a comprehensive overview of the experimental procedure.

Following the general preparation, but before induction of cardiac arrest, animals were randomized to either receive 20 ppm of iNO (n = 10) or nitrogen (n = 10, control), using the closed envelope method. The respective gas was applied using a human-grade intensive care respirator (Servo Ventilator 300A, Siemens, Munich, Germany).

Cardiac arrest was electrically induced, and confirmed by the onset of VF and a rapid decrease in mean arterial pressure (MAP). Simultaneously, mechanical ventilation was discontinued and the animal left untreated for 10 minutes. Then, iCPR-treatment was initiated by activating the previously implanted pLVAD at the maximum possible flow. No additional chest compressions were delivered during any phase of the experiment. To achieve maximum flow, the Impella controller was set to Auto-mode, in which the software automatically determines the maximum flow capacity by evaluating the motor current to identify wall suction. Simultaneously with the start of the pLVAD, ventilation was restarted with an inspired oxygen fraction (FiO_2_) of 1.0, blended with either 20 ppm of iNO or nitrogen, before animals received a bolus dose of 30 μg/kg adrenaline and Ringer’s solution at a rate of 100 mL/minute. A second dose of adrenaline was administered 4 minutes and 30 seconds after the start of resuscitation.

After six minutes of iCPR, defibrillation was attempted with up to two 200-J biphasic waveform shocks (M-Series CCT; Zoll Medical Corporation, Chelmsford, MA, USA). If an organized rhythm with an MAP >60 mmHg persisted for 5 minutes, the animal was regarded as successfully resuscitated. If VF was not successfully reversed, one minute of iCPR preceded the delivery of another sequence of up to two shocks.

After successful resuscitation, anesthesia was continued, and animals were monitored for 6 hours. In the iNO group, animals were ventilated with NO at 20 ppm for up to 5 hours after ROSC. Thirty minutes following ROSC, FiO2 was reduced to 0.3 in both groups. After the 6-hour observation period, anesthesia was stopped and animals weaned from the respirator as previously described in detail [2]. Following the removal of all foreign materials, animals were brought to their cages and clinically observed for up to 5 days.

### Measurements

Blood pressure, heart rate, and EKG (electrocardiogram) readings were continuously measured and digitally recorded. Pulmonary artery and pulmonary capillary wedge pressure (PCWP) were measured using a hexalumen Swan-Ganz catheter (744HF75, Edwards Lifesciences, Irvine, CA, USA) and a Vigilance monitor. Cerebral oxymetry was performed and tracked using the INVOS 5100c cerebral oxymeter and appropriate INVOS cerebral oxymetry adult sensors (both Covidien, Dublin, Ireland). Coronary perfusion pressure (CPP) was calculated by subtracting the mid-diastolic right atrial pressure from the mid-diastolic aortic pressure [13]. However, due to the non-pulsatile flow generated by the pLVAD, CPP was calculated by subtracting the mean right atrial pressure from mean aortic pressure during CPR. Pulmonary vascular resistance was calculated using equation (1) as follows:

1 $$ \frac{80\times \left( MPAP- PCWP\right)}{Cardiac\kern0.35em  Output}, $$

where MPAP is the mean pulmonary artery pressure and PCWP the pulmonary capillary wedge pressure.

However, during iCPR, PCWP and cardiac output were not available for technical reasons. PCWP was therefore omitted and cardiac output replaced by pLVAD flow, resulting in equation (2). Calculating pulmonary vascular resistance (PVR) without accounting for PCWP is frequently referred to as total PVR [14]. Equation (2) is as follows:

2 $$ \frac{80\times (MPAP)}{iCPR\kern0.5em  flow} $$

The arterial oxygen and carbon dioxide tension (P_a_O_2_ and P_a_CO_2_, respectively), blood glucose levels, and lactate levels were measured using a point-of-care blood gas analyzer (ABL 510; Radiometer, Copenhagen, Denmark). Blood samples were obtained at baseline (i.e., 5 minutes before cardiac arrest) and every hour for 6 hours after ROSC. At the same time points, serum samples to determine astroglial S-100 protein levels (to quantify neuronal cell death) were obtained and promptly frozen to allow for measurements at a later time using a commercially available ELISA kit (YK150, BioTrend, Cologne, Germany).

### Post-arrest care

Following the resuscitation procedure and weaning from the respirator, animals were brought to their cages and monitored permanently by research staff. Analgesia was provided by an intramuscular injection of 0.1 mg/kg buprenorphine if tachypnea (>20/minutes) was observed or an animal seemed agitated. Chow and water were offered by gentle spoon-feeding in cases where animals exhibited inadequate food intake. If an animal was not able to swallow, Ringer’s solution was administered intravenously at a rate of 10 mL/kg/h. Animals that did not improve with this protocol and were not able to stand up and walk within 48 hours were euthanized by intravenous injection of a lethal dose of pentobarbital.

Euthanized animals were perfused with 4 % paraformaldehyde and brains carefully harvested and histopathologically analyzed as described in [12]. All animals underwent systematic necropsy of the thoracic and abdominal cavities to identify injuries to the chest or thoracic or visceral organs.

### Neurological testing

On each day post-arrest, animals were evaluated using overall performance categories (OPC), as described in previous studies [10, 11]. In brief, the test consists of five items representing the degree of impairment as follows: OPC 1, normal, no obvious neurologic damage; OPC 2, moderate disability, animals being conscious and aware, standing but unable to walk; OPC 3, severe disability, animals being neither fully aware nor unconscious, but with reaction to pain and auditory stimuli, not able to stand or walk; OPC 4, coma; and OPC 5, death or brain death. In animals receiving buprenorphine as part of the post-arrest care protocol, an adequate interval (>4 hours) was allowed to elapse before neurological testing was performed.

In addition, animals were challenged with a visual spatial memory task, described previously in detail in [10]. In this test, animals are trained individually to obtain food from three feeding troughs in an area separated from their housing. Following acclimatization with the setting, the lids of two of the containers are locked, and the animal is expected to learn that one trough can still be opened, and to remember this very container. To quantify the performance, time elapsed from entering the separated area until food is obtained is recorded.

### Statistical analysis

All data are expressed as the mean ± SD unless stated otherwise. Normal distribution of the data was confirmed using the Kolmogorov-Smirnov test. For group comparisons of continuous variables, repeated measures analysis of variance (ANOVA) was employed followed by the pairwise Student’s t test at given time points, adjusted for multiple comparisons by Bonferroni’s method in cases where significant differences were observed. Where appropriate, Fisher's exact test and the Mann–Whitney *U* test was performed to compare categorical variables. In all cases, *p* ≤0.05 was considered to indicate statistical significance.

## Results

### Inhaled NO increased pLVAD flow and coronary perfusion pressure during iCPR

Hemodynamics and blood gas results did not differ between groups before iCPR was initiated (Table 1). Group assignment did not influence time to ROSC (iNO vs. Control: 16.5 ± 0.7 vs. minutes, *p* = 0.214), amount of adrenaline given (2.2 ± 0.6 vs 2.0 ± 0.1 mg, *p* = 0.382) or the number of shocks (2.6 ± 1.8 vs 3.5 ± 2.6, *p* = 0.387).

**Table 1:** Hemodynamics and blood gas data

		BL	PR 10	PR 30	PR 60	PR 360
		iNO n = 10	iNO n = 10	iNO n = 10	iNO n = 10	iNO n = 10
		Control n = 10	Control n = 10	Control n = 10	Control n = 10	Control n = 10
HR (bpm)	iNO	89 ± 16	156 ± 47	130 ± 56	111 ± 22	104 ± 19
	Cont	81 ± 12	192 ± 33	164 ± 33	124 ± 24	102 ± 23
MAP (mmHg)	iNO	91 ± 13	89 ± 8*	72 ± 15	70 ± 14	83 ± 6*
	Cont	94 ± 9	104 ± 14	81 ± 12	78 ± 9	92 ± 10
CO (L/minute)	iNO	5.0 ± 2.1	7.8 ± 2.5	4.3 ± 1.8	3.8 ± 1.5	4.6 ± 0.9
	Cont	5.2 ± 0.9	9.0 ± 1.8	5.4 ± 1.4	3.6 ± 0.7	6.0 ± 2.2
MPAP (mmHg)	iNO	14 ± 44	15 ± 4*	13 ± 3	12 ± 3*	19 ± 5
	Cont	16 ± 4	21 ± 3	16 ± 4	18 ± 4	20 ± 4
P_a_O_2_ (mmHg)	iNO	139 ± 5	434 ± 92*	329 ± 199	136 ± 6	123 ± 18
	Cont	140 ± 6	504 ± 68	454 ± 174	138 ± 8	135 ± 13
P_a_CO_2_ (mmHg)	iNO	42 ± 4	40 ± 2	38 ± 3	39 ± 4	43 ± 4
	Cont	41 ± 3	44 ± 5	39 ± 4	38 ± 4	42 ± 5
Lactate (mmol/L)	iNO	1.4 ± 0.7	6.8 ± 1.4	6.9 ± 1.5	5.9 ± 1.9	0.7 ± 0.2
	Cont	1.4 ± 0.2	7.5 ± 1.2	7.3 ± 1.4	5.7 ± 1.1	1.0 ± 0.5
Glucose (mmol/L)	iNO	118 ± 16	277 ± 19	252 ± 25	234 ± 31	117 ± 17*
	Cont	127 ± 13	294 ± 33	274 ± 24	251 ± 29	132 ± 12
pH	iNO	7.47 ± 0.02	7.35 ± 0.03*	7.40 ± 0.06	7.41 ± 0.06	7.46 ± 0.03
	Cont	7.47 ± 0.04	7.30 ± 0.04	7.37 ± 0.04	7.42 ± 0.04	7.47 ± 0.04

Hemodynamic and blood gas data in 20 pigs, treated either with 20 ppm of inhaled nitric oxide (iNO; n = 10) or 0 ppm iNO (Control; n = 10) at baseline (BL) or 10 (PR10), 30 (PR30), 60 (PR60), or 360 (PR360) minutes after return of spontaneous circulation. Results are presented as mean ± SD; *p ≤0.05 for iNO vs Control. *HR* heart rate, *MAP* mean arterial pressure, *CO* cardiac output, *MPAP* mean pulmonary artery pressure, *P*_*a*_ *O*_*2*_ arterial oxygen tension, *P*_*a*_ *CO*_*2*_ arterial carbon dioxide tension

**Fig. 2:**
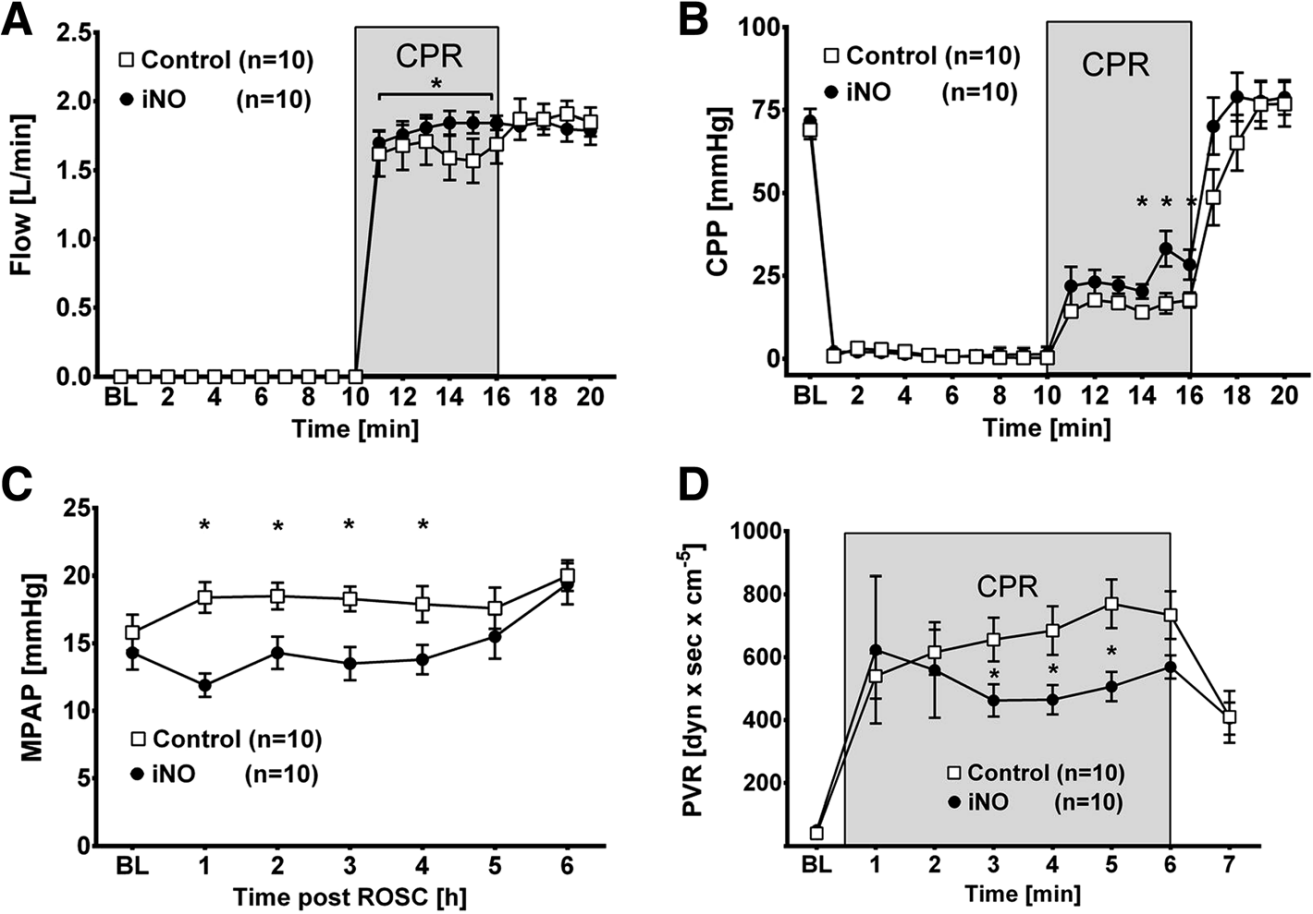
Inhaled nitric oxide (*iNO*) impacts hemodynamics during and after cardiac arrest. Comparison of animals treated with 20 ppm iNO (n = 10) or Control (n = 10). Mean ± standard error of the mean: *p ≤0.05, comparing iNO to Control. *BL* baseline (i.e., 5 minutes prior to cardiac arrest), *CPR* cardiopulmonary resuscitation. **a** Pump flow of the intravascular cardiopulmonary resuscitation device. **b** Calculated coronary perfusion pressure (*CPP*). **c** Mean pulmonary artery pressure (*MPAP*) measured at BL and 1, 2, 3, 4, 5, and 6 hours after return of spontaneous circulation (*ROSC*). **d** Calculated pulmonary vascular resistance (PVR) before (BL), during (1–6 minutes), and following cardiac arrest and resuscitation (7 minutes). Time indicates period since start of CPR. The 10-minute period of untreated ventricular fibrillation preceding CPR was omitted for clarity

During iCPR, iNO treatment allowed for higher average pump flow compared to control (1.81 ± 0.30 vs 1.64 ± 0.51 L/minute, *p* <0.001, see Fig. 2a), translating into higher CPP values during the 6 minutes of iCPR before the first defibrillation was attempted (25 ± 13 vs 16 ± 6 mmHg, p = 0.002, Fig. 2b). iNO-treatment did not affect MPAP during iCPR (12 ± 6 vs 13 ± 3 mmHg, *p* = 0.284). However, we observed significantly lower MPAP in animals that breathed NO than those breathing nitrogen as soon as 3 minutes after the ROSC (17 ± 3 vs 23 ± 5 mmHg, *p* = 0.003). This difference in MPAP continued until iNO was stopped at 5 hours after ROSC (Fig. 2c). Calculated total PVR appeared to be effectively lowered, beginning 3 minutes after the start of iNO treatment during CPR (Fig. 2d).

### NO breathing increased cerebral oxygenation without increasing cardiac output after ROSC

**Fig. 3:**
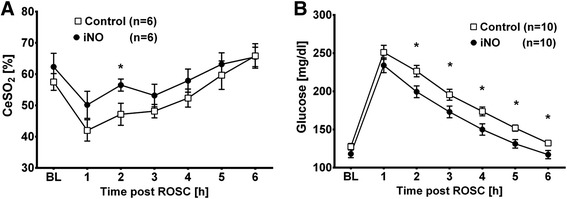
Inhaled nitric oxide (*iNO*) improves cerebral oxygenation and suppresses the increase of glucose after return of spontaneous circulation (*ROSC*). Comparison of animals treated with 20 ppm iNO (n = 10) or Control (n = 10). Mean ± standard error of the mean: *p ≤0.05, comparing iNO to Control. *BL* baseline (i.e., 5 minutes prior to cardiac arrest). **a** Cerebral oxygenation (*CeSO*_*2*_) was estimated using transcutaneous spectroscopy using near-infrared light. Due to limited availability during experiments, values were estimated in 6 animals per group. **b** Serum glucose levels before and after cardiac arrest and resuscitation

Breathing NO increased cerebral oxygenation at 2 hours after ROSC (56.5 ± 4.7 vs 47.2 ± 8.6 %, *p* = 0.043; Fig. 3a). End-tidal CO_2_ values as a measure of the quality of circulation trended towards higher numbers with iNO treatment than in controls. However, this was not statistically significant at any time point during CPR. Inhaled NO did not affect cardiac output in the post-ROSC period either (Table 1).

### Inhaled NO suppressed the increase of glucose after ROSC

Blood glucose levels were not different at baseline and up to one hour after ROSC. However, animals treated with iNO had significantly lower blood glucose values compared to controls, starting from 2 hours after the start of iNO until 6 hours after the CPR procedure (Fig. 3b).

### NO breathing improved neurological outcome after cardiac arrest

**Fig. 4:**
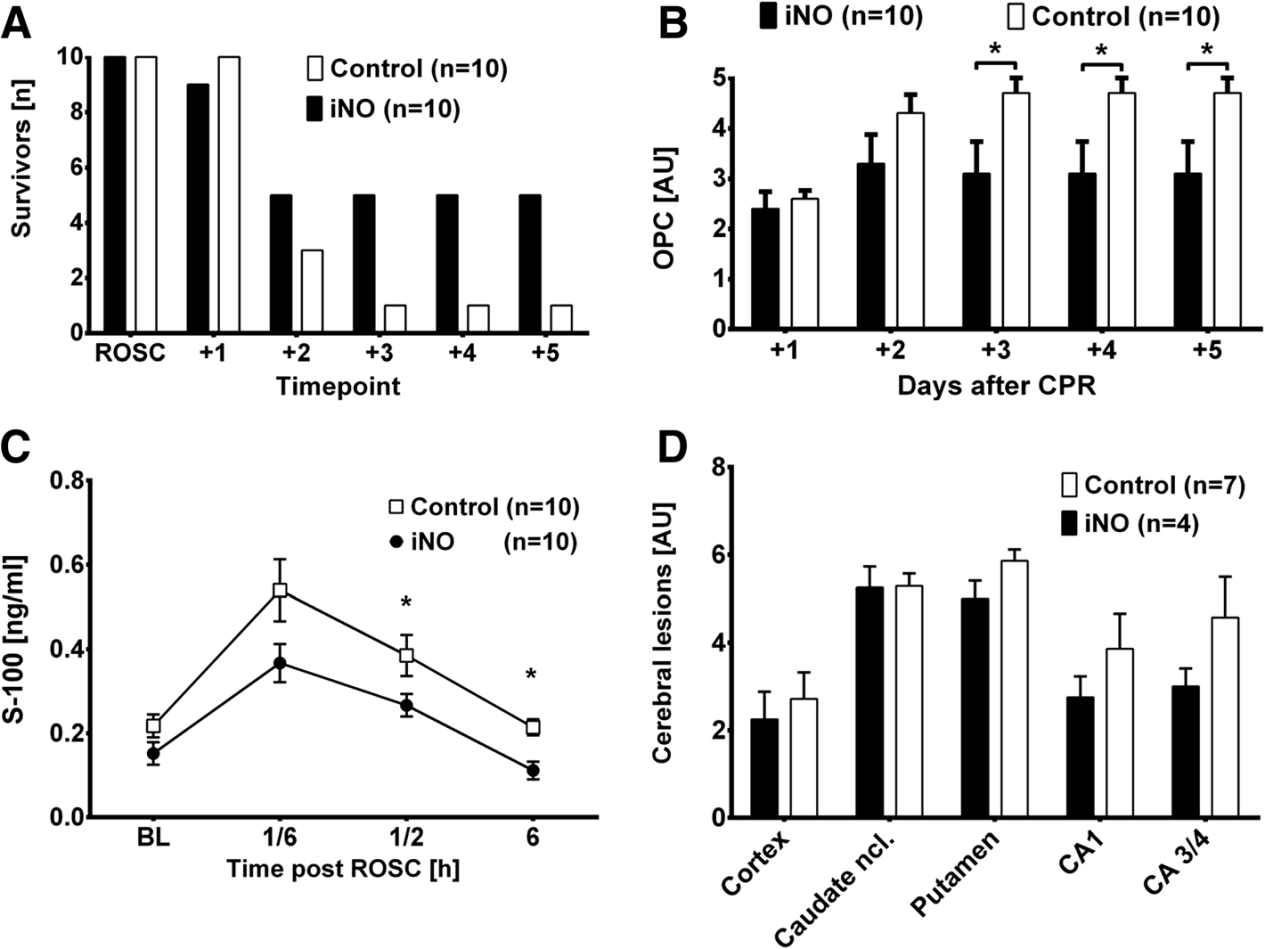
Inhaled nitric oxide (*iNO*) impacts the outcome after cardiac arrest. Comparison of animals treated with 20 ppm iNO (n = 10) or Control (n = 10). Mean ± standard error of the mean: *p ≤0.05, comparing iNO to Control. *ROSC* return of spontaneous circulation, *BL* baseline (i.e., 5 minutes prior to cardiac arrest), *AU* arbitrary units. **a** Number of animals that achieved ROSC or were alive on days 1 (*+1*), 2 (*+2*), 3 (*+3*), 4 (*+4*) or 5 (*+5*) post cardiopulmonary resuscitation (*CPR*). **b** Overall performance categories (*OPC*): OPC 1, normal, no obvious neurologic damage; OPC 2, moderate disability, animals being conscious and aware, standing but unable to walk; OPC 3, severe disability; animals being neither fully aware nor unconscious, but with reaction to pain and auditory stimuli, not able to stand or walk; OPC 4, coma; OPC 5, death or brain death. **c** S-100 protein levels measured at *BL*, 10 minutes (*1/6*), 30 minutes (*1/2*), or 6 hours after ROSC. S-100 serum levels were employed as an indicator of the degree of cerebral ischemia-reperfusion injury. **d** Results from the blinded neurohistopathological evaluation in animals euthanized 24 hours after ROSC due to their inability to rise

All animals in both groups achieved ROSC. However, while five animals that breathed NO survived until day 5 post CPR, only one control animal survived until the end of the observation period (*p* = 0.141, Fig. 4a). While three iNO-treated animals were able to participate in the spatial memory task on the first day post CPR, only one of the controls were able to do so (Table 2). Nine control and five iNO-treated animals had to be euthanized prematurely due to severe neurological deficits. This translated into significantly better score values for iNO-treated animals in the outcome assessment with OPC on days 3−5 post CPR (3 ± 2 vs 5 ± 1, *p* = 0.036 on days 3−5; Fig. 4b).

**Table 2:** Spatial memory task

	iNO			Control		
	Alive (n)	Testable (n)	Time (s)	Alive (n)	Testable (n)	Time (s)
Day						
−5	10	10	475 ± 233	10	10	498 ± 188
−4	10	10	338 ± 249	10	10	162 ± 251
−3	10	10	35 ± 36	10	10	141 ± 261
−2	10	10	9 ± 5	10	10	74 ± 197
−1	10	10	7 ± 2	10	10	13 ± 11
CPR						
+1	9	3	406 ± 336	10	0	−
+2	5	3	402 ± 344	3	1	600 ± 0
+3	5	3	403 ± 341	1	1	600 ± 0
+4	5	3	119 ± 115	1	1	600 ± 0
+5	5	3	91 ± 126	1	1	600 ± 0

Performance of the tested animals in a spatial memory task before (Days -5 to -1) and after (Days +1 to +5) cardiac arrest and cardiopulmonary resuscitation (CPR). Time in seconds is provided as mean ± SD. The ability to participate in the test was defined as the ability to stand and walk in conjunction with physiologic searching behavior. Up to 600 seconds were allowed to complete the test. *iNO* inhaled nitric oxide

The favorable neurological outcome was associated with lower S-100 serum levels in iNO-treated animals, detected as early as 30 minutes post ROSC (0.26 ± 0.09 vs 0.38 ± 0.15 ng/mL, *p* = 0.048; Fig. 4c).

Neurohistopathological examination of vulnerable cerebral structures prone to ischemia-reperfusion injury trended towards fewer cerebral lesions in iNO-treated vs control animals euthanized on day 1 following the CPR procedure (neocortex: 2.5 ± 1.7 vs 3.7 ± 2.3 AU, *p* = 0.192 ; archicortex 5.8 ± 1.5 vs 8.4 ± 4.5 AU, *p* = 0.142 ; striatum 10.3 ± 1.7 vs 11.1 ± 1.2 AU, p = 0.168; Fig. 4d). Furthermore, the single control animal surviving until day 5 exhibited greater neuroistopathological damage than did the five iNO-treated animals that survived thus far (neocortex: 4.6 ± 1.7 vs 7.0 AU; archicortex 10.2 ± 4.8 vs 13.0 AU; striatum 12.6 ± 6.5 vs 12.0 AU). Of note, the neurohistopathological damage scores appeared to rise over time in both groups, suggesting a secondary injury of the cerebral tissue following the acute ischemic insult during cardiac arrest. Systematic necropsy of the animals revealed no macroscopic trauma to heart, valves, or major vessels.

## Discussion

Here we show for the first time, that iNO enhances transpulmonary blood flow during resuscitation with a percutaneous axial left ventricular assist device. Improved cerebral perfusion during iCPR and after ROSC was associated with improved cerebral oxygenation and with lower serum levels of S-100 protein. In addition we observed a significant impact of iNO on glucose serum levels. These beneficial hemodynamic and metabolic effects translated into improved clinical outcomes in the first 5 days following resuscitation.

### Improving hemodynamics with iNO during extracorporeal life support and iCPR

Recently, Lovich and colleagues have shown that iNO is capable of improving the maximum flow capacity of a surgically implanted extracorporeal LVAD in a porcine model of hypoxemia-induced pulmonary hypertension and right ventricular failure [8]. In their study, 20 ppm of iNO increased the maximum achievable pump speed, flow, cardiac output, and left ventricular diameter. iNO reduced pulmonary vascular resistance, right ventricular (RV) afterload and thereby facilitated transpulmonary blood flow. These observations are consistent with previous findings in a small randomized double-blind clinical trial in which iNO was shown to decrease pulmonary arterial pressure (PAP) and increase LVAD flow [15]. For the first time, our study extends the concept of improving transpulmonary blood flow with iNO to the condition of LVAD support during CPR after prolonged cardiac arrest.

Pulmonary artery pressure is known to be elevated during conventional cardiac arrest and resuscitation, with elevated pulmonary vascular resistance being the primary cause [16, 17]. The intermittent high intrathoracic pressure during external chest compressions in concert with hypoxic pulmonary vasoconstriction may limit right-to-left transpulmonary bloodflow during CPR. We have previously shown that omitting chest compressions during iCPR reduces pulmonary blood pressure, but still does not solve the problem of limited transpulmonary blood flow [2]. As iNO is a potent local vasodilator, one could expect a further lowering of the mean pulmonary artery pressure during CPR. However, this was not the case in our investigation, as PAP was only reduced by iNO after ROSC, but not during iCPR. In contrast, we saw a pronounced lowering of pulmonary vascular resistance during iCPR. As the pLVAD controller increased flow as soon as the pulmonary vascular tone was lowered, MPAP remained constant. The improved pLVAD flow during iCPR then translated into higher mean arterial pressures, which in turn led to improved calculated CPP during resuscitation. This observation suggests that, when performing iCPR, MPAP may not be a reliable indicator of effective pulmonary vasodilation.

### Regional and systemic effects of iNO during and after cardiac arrest

Alterations in organ perfusion by iNO do not appear to be the sole factor for influencing the clinical outcome, because iNO did not affect serum lactate levels, a marker for poor tissue perfusion, at any point during the investigation. Nonetheless, we still observed a pronounced reduction in S-100 serum protein, a proposed marker for the degree of cerebral injury [18, 19]. Besides its beneficial effects on iCPR performance and hemodynamics, iNO-treated animals exhibited a significantly better clinical outcome compared to control animals in our study. These results corroborate findings from others, and for the first time extend them to a large-animal model of cardiac arrest and CPR. Minamishima and colleagues found that in mice, breathing NO starting one hour after ROSC markedly improved long-term neurological outcomes and survival after 7.5 minutes of cardiac arrest, without causing systemic hypotension [20]. Neuroprotective effects of iNO have been subsequently confirmed by several groups that showed that inhaled NO decreased brain stroke volume in mice, rats, and sheep [21–23]. Besides its neuroprotective effects, several researches have reported cardioprotective effects of iNO in mice and pigs [24–26]. The ability of iNO to reduce I/R (ischemia/reperfusion) injury was subsequently reproduced in proof-of-principle human studies [7, 27]. Hence, our current study provides further support for the organoprotective properties of iNO at clinically relevant concentrations, when given during and after cardiac arrest.

The mechanisms through which iNO exerts its protective effects in remote organs are incompletely understood. Inhaled NO may exert systemic effects via interaction with circulating bone-marrow-derived cells (e.g., leukocytes) as they transit the lungs. Alternatively, some NO, once inhaled, may escape scavenging by hemoglobin and be converted to relatively stable NO-metabolites (e.g., nitrite, S-nitrosothiols) that can regenerate NO in the periphery and directly protect neurons [28, 29]. In the current study, we observed that iNO prevented cardiac arrest-induced increase of blood glucose levels. Elevated glucose serum levels seen in survivors of cardiac arrest have been identified as a predictor of adverse clinical outcomes following resuscitation [30–33]. While glucose serves as a marker of post-aggression metabolism and therefore, the degree of ischemia-reperfusion injury, glucose per se may harm the organism when reaching excessively high levels. Nonetheless, how iNO prevented the rise of glucose levels after CA (Cardiac arrest) is currently unknown. Mechanisms responsible for the protective effects of iNO in remote organs remain to be determined in future studies.

In this investigation, we observed significantly lower serum levels of S-100 protein in iNO-treated animals, suggesting neuroprotective effects of iNO. Nonetheless, although we saw improved clinical outcomes in iNO-treated animals, we failed to detect histological evidence that supports iNO-mediated neuroprotection. This is likely to be due to the limited sample size and limited number of animals surviving until day 5 of the follow up period. It is also likely that we failed to detect significant improvement in survival during the observation period due to the small sample size and high mortality rate.

### Limitations

There are limitations when interpreting the results of our study: first, iCPR is a technique not frequently used in clinical emergency medicine and may not produce the same hemodynamic effects as chest compression CPR. Therefore, our results may not be readily transferable to settings in which chest compressions are used to perform CPR. On the other hand, use of the iCPR device enabled standardization of the quality of CPR, thus revealing the important beneficial effects of iNO in our unique model of cardiac arrest. This study was conducted in healthy, juvenile animals. Therefore, the effects of iNO may be different in human cardiac arrest patients with multiple comorbidities. While our current study was not designed to examine the optimal dose or timing of iNO administration, it is notable that the response to iNO to prevent I/R injury is similar across multiple species including man [7, 24, 25]. Further studies examining the effects of iNO during chest compression CPR, and optimal dose and timing of administration, are therefore warranted.

## Conclusions

In summary, the current study showed that iNO augments pLVAD flow during iCPR, leading to better hemodynamics and improved clinical outcomes in a large-animal model of cardiac arrest. The beneficial effects of iNO may not solely depend on the improved pLVAD flow, but may also be mediated via direct neuroprotective effects of iNO in the post-arrest period. Currently, there is no pharmacological therapy that is known to improve outcomes after cardiac arrest. Along with accumulating evidence of the protective effects of iNO after I/R injury including cardiac arrest, this first large-animal study should provide a solid foundation to translate this unique therapy to improve outcomes after cardiac arrest in patients.

## Key messages

- iNO facilitates transpulmonary blood flow during iCPR
- Using 20 ppm iNO improves clinical outcomes after CPR in pigs
- Optimal dosing and timing needs to be verified in time-course and dose-response experiments

## Abbreviations

ANOVA: analysis of variance
AU: arbitrary units
BL: baseline
CA: cardiac arrest
CeSO_2_: cerebral oxygenation
CO: cardiac output
CO_2_: carbon dioxide
CPP: coronary perfusion pressure
CPR: cardiopulmonary resuscitation
ECLS: extracorporeal life support
ECMO: extracorporeal membrane oxygenation
eCPR: extracorporeal CPR using ECMO
ELISA: enzyme-linked immunosorbent assay
etCO_2_: end-tidal carbon dioxide
F_i_O_2_: inspired oxygen fraction
HR: heart rate
iCPR: intravascular cardiopulmonary resuscitation
iNO: inhaled nitric oxide
LANUV: Landesamt für Natur, Umwelt und Verbraucherschutz NRW
MAP: mean arterial pressure
mmHg: millimeter of mercury
MPAP: mean pulmonary artery pressure
MTH: mild therapeutic hypothermia
O_2_: oxygen
OPC: overall performance categories
P_a_CO_2_: arterial carbon dioxide tension
P_a_O_2_: arterial oxygen tension
PAP: pulmonary artery pressure
pCO_2_: carbon dioxide tension
PCWP: pulmonary capillary wedge pressure
ppm: parts per million
PVR: pulmonary vascular resistance
ROSC: return of spontaneous circulation
SD: standard deviation
SEM: standard error of the mean
sGC: soluble guanylate cyclase
VF: ventricular fibrillation
